# A chiari network mimicking a cystic structure

**DOI:** 10.1007/s12471-014-0621-1

**Published:** 2014-10-29

**Authors:** J. Walpot, G. Sahin-Arpaci, M. Sadreddini

**Affiliations:** Department of Cardiology, Admiraal De Ruyter Hospital, Goes & Vlissingen, Koudekerkseweg 88, PO Box 3200, 4380 DD Vlissingen, the Netherlands

**Keywords:** Chiari network, Cystic structure, Echocardiography

## Abstract

The Chiari network is a net-like mobile structure, occasionally encountered near the entrance of the inferior vena cava in the right atrium. Due to its fenestration, the Chiari network does not cause flow obstruction of the blood. The Chiari network is usually an incidental finding with no further clinical consequences. We report an unusual presentation of a Chiari network, mimicking a right atrial oscillating cystic mass attached to the interatrial septum by a thin stalk.

**Electronic supplementary material:**

The online version of this article (doi:10.1007/s12471-014-0621-1) contains supplementary material, which is available to authorized users.

## Image

**Fig. 1:**
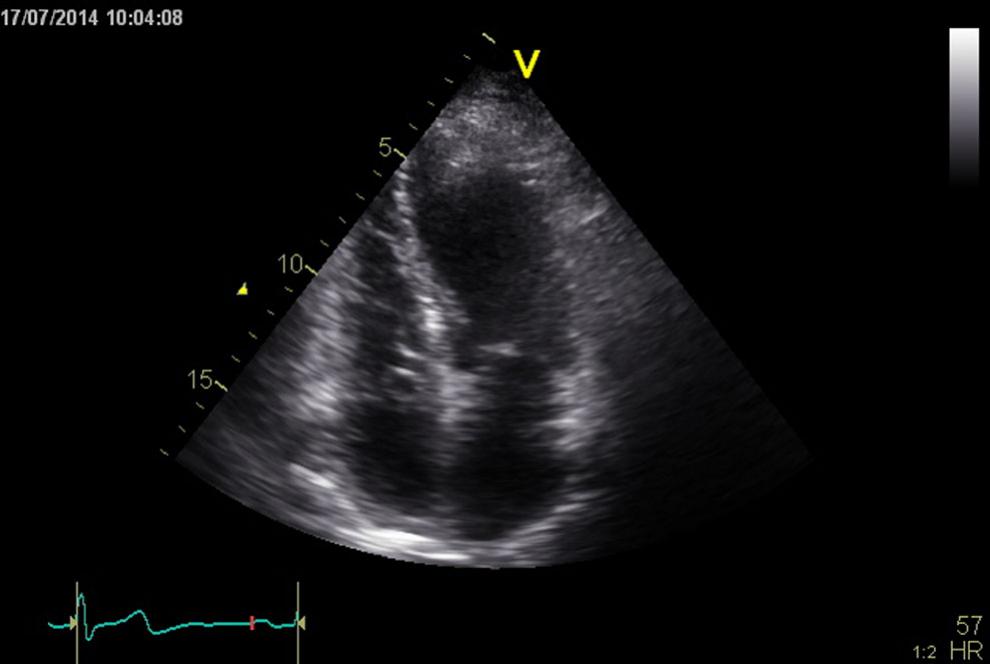
The TTE four-chamber view demonstrates a cystic mass attached to the interatrial septum by a thin stalk in the right atrium. (See also Video 1)

A 66-year-old man suffered from a cerebrovascular insult. The ECG showed sinus rhythm. Holter tape recording did not demonstrate paroxysmal atrial fibrillation. The transthoracic echocardiographic (TTE) study showed a preserved left and right ventricular systolic function. An oscillating cystic structure, attached to the interatrial septum (IAS) by a thin stalk, was seen in the right atrium. (Fig. 1, Video 1)

**Fig. 2:**
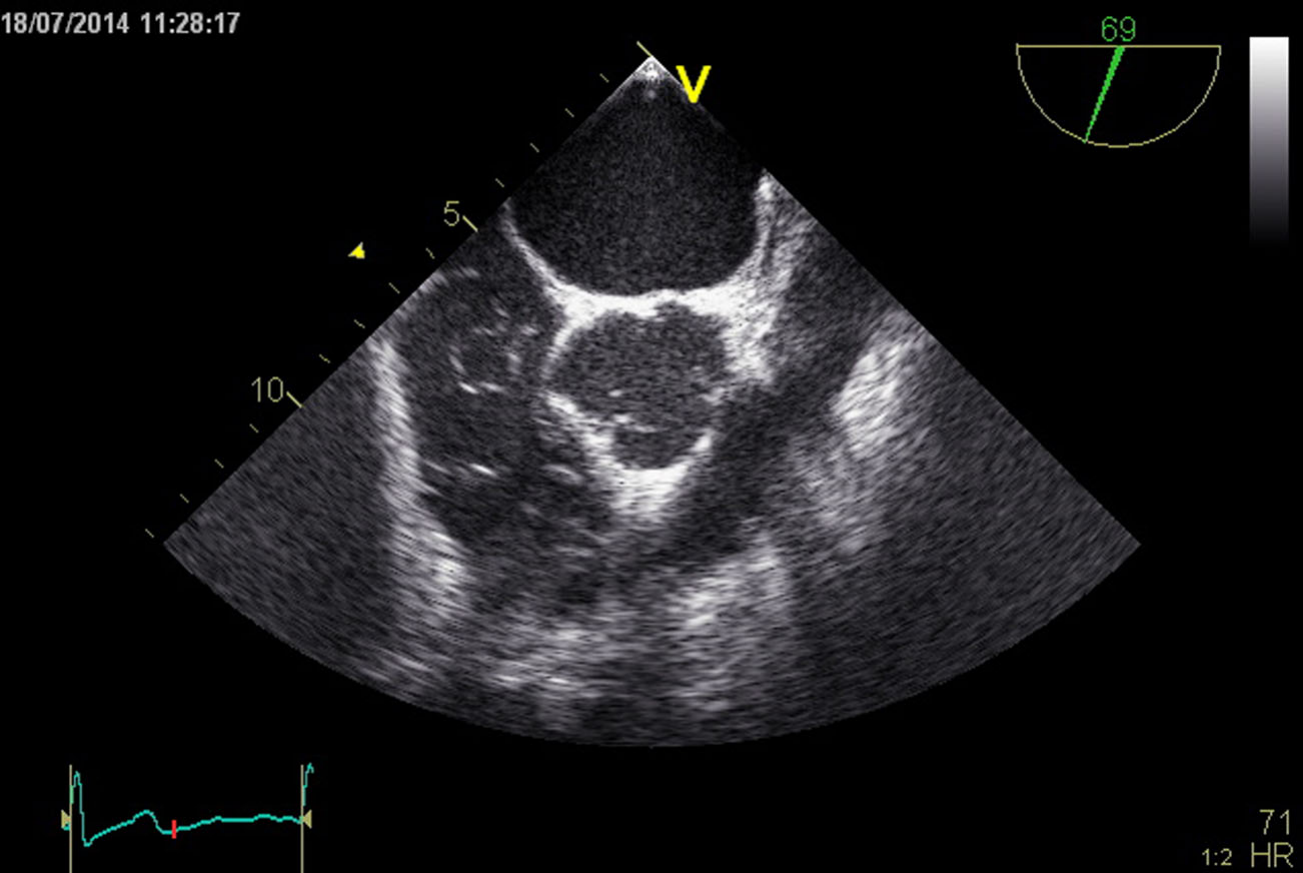
TEE image at 69° shows the large Chiari network in the right atrium. (See also Video 2)

Further analysis by TEE revealed an elongated Eustachian valve and a large mobile Chiari network. (Fig. 2, Video 2 and 3) The Chiari network was identified as the structure causing the pseudocystic image, which had been seen on TTE. Intravenous injections of agitated colloid solution could not demonstrate a punched out lesion consistent with a cyst. Furthermore, a patent foramen ovale was excluded.

The Chiari network, a remnant of the right valve of the sinus venosus, is a mobile net-like structure occasionally seen in the right atrium near the opening of the inferior vena cava and coronary sinus. Due to its fenestration, the Chiari network does not cause flow obstruction of the blood. It is usually an incidental finding with a reported prevalence ranging from 2 to 13.6 % in echocardiography studies and autopsy series [1–3]

Prevention of pulmonary embolism due to entrapment of thrombi, originating from the deep venous system, in the Chiari network has occasionally been reported [4] A few medical case reports have described entrapment of catheters in the Chiari network during invasive procedures [5] Rarely, the Chiari network is identified as the site of infective endocarditis.

## Electronic supplementary material

Below is the link to the electronic supplementary material.

Video 1 TTE four-chamber view cine loop demonstrates an oscillating cystic mass attached to the interatrial septum by a thin stalk in the right atrium. (AVI 5993 kb)

### Electronic supplementary material

Video 2 TEE cine loop at 30° shows the large mobile Chiari network in the right atrium, creating pseudocystic images throughout the heart cycle. (WMV 1148 kb)

### Electronic supplementary material

Video 3 TEE biatrial view at 120°. The cine loop demonstrates the elongated Eustachian valve and the Chiari network, presenting as cystic structure in this imaging plane. (WMV 1070 kb)

### Electronic supplementary material

Video 4 TEE cine loop after intravenous injection of agitated colloid solution. Note the complete opacification of the right atrium, excluding a cyst. Right-to-left shunting through a patent foramen ovale was also excluded. (WMV 1184 kb)

## References

[1] Werner JA, Cheitlin MD, Gross BW (1981). Echocardiographic appearance of the Chiari network: differentiation from right heart pathology. Circulation.

[2] Bhatnagar KP, Nettleton GS, Campbell FR, Wagner CE, Kuwabara N, Muresian H (2006). Chiari anomalies in the human right atrium. Clin Anat.

[3] Islam AK, Sayami LA, Zaman S (2013). Chiari network: A case report and brief overview. J Saudi Heart Assoc.

[4] Zuzana M, Petr W, Dana B, Martin P, Hana L, Dana K, Miroslav K, Ludmila K, Jan K. An embolus in the right atrium caught in the Chiari network and resistant to thrombolysis. BMJ Case Rep. 2010 Sep 19;2010. pii: bcr0920081019. 10.1136/bcr.09.2008.1019 PMC3038023 22791494

[5] Dissmann R, Schröder J, Völler H, Behrens S (2006). Entrapment of pacemaker lead by a large netlike Eustachian valve within the right atrium. Clin Res Cardiol.

